# The Influence of Hydroxyapatite Nanoparticle Morphology on Embryonic Development in a Zebrafish Exposure Model

**DOI:** 10.3390/nano7040089

**Published:** 2017-04-22

**Authors:** Shiuli Pujari-Palmer, Xi Lu, Marjam Karlsson Ott

**Affiliations:** Department of Engineering Science, Applied Materials Science, Uppsala University, 75106 Uppsala, Sweden; shiuli.pujaripalmer@maiiadiagnostics.com (S.P.-P); cqluxi@gmail.com (X.L)

**Keywords:** nanoparticle morphology, hydroxyapatite, zebrafish development

## Abstract

Nanomaterials are used in many different industries such as cosmetics, food, clothing, and electronics. There is increasing concern that exposure to nanoparticles (NPs) during pregnancy can adversely affect fetal development. It is well known that the size, charge, and chemistry of a nanoparticle can modulate embryological development. The role that particle morphology plays on early development, however, is still widely unknown. The present study aims to investigate the effect of hydroxyapatite nanoparticle (HANP) morphology on embryological development in a zebrafish exposure model. Four distinct HANP morphologies (dots, long rods, sheets, and fibers) were fabricated and characterized. Zebrafish embryos were exposed to HANPs (0–100 mg/L), and viability and developmental deformities were evaluated for up to 5 days post-fertilization (dpf). Malformations such as pericardial edema and axial curvature were apparent in embryos as early as 1 dpf, following exposure to the dot and fiber particles, and developed in embryos by 3 dpf in the sheet and long rod particle groups. Minimal death was observed in response to dot, long rod, and sheet particles (≤25%), while fiber particles induced overwhelming toxicity (≤60%) after 1 dpf, and complete toxicity during all subsequent time points. Collectively, these results suggest that nanoparticle morphology can significantly impact embryological development and should be a required consideration when designing nanomaterials for commercial use.

## 1. Introduction

The use of nanoparticles (NPs) in many common household items has increased the likelihood and intensity of human exposure, e.g., silver NPs found in clothing and sheets, titanium dioxide NPs found in cosmetics and sunscreen products, and carbon NPs found in bikes and other transportation vehicles [1]. Although nanomaterials have attractive and unique features, there is increasing evidence that these features can also make them reactive, resulting in potential health risks [2]. There are many routes of exposure for nanomaterials; nanomaterials can enter the body via inhalation, skin penetration, or ingestion [3]. Nanomaterial exposure can also occur following the implantation of a biomaterial within the body. A popular orthopedic biomaterial for bone and dental regenerative applications is calcium phosphate ceramics, since it has an inorganic mineral composition, physical characteristics, and a porosity similar to human bone [4,5]. Within the calcium phosphate family, hydroxyapatite (HA) is most similar to mature bone. HA is often used as a bone filler, and as a coating for metal implants to improve fixation of the implant to bone tissue [6]. Though HA is promising for clinical use [7,8], some reports have suggested that HA coatings can produce nanoparticulate debris, causing toxicity and inflammation in the body [9,10]. In pregnant mice it has been shown that NPs cross the placenta, accumulate in the fetus [11], restrict blood flow, reduce placenta size, and cause fetal growth restriction, eventually causing death. The effect of particle size, chemistry, and charge on early development has been extensively investigated [12,13,14], in contrast to the effects of particle morphology, which has not been studied conclusively.

In order to evaluate the effect of NP morphology on fetal development, we have chosen to use a zebrafish exposure model. Zebrafish are small in size, reproduce and develop quickly, have transparent embryos, etc., making it a very popular model to use in the fields of toxicology and biomedical research. In addition, zebrafish and human genomes share 75% homology, i.e., [15] the cardiovascular, nervous, inflammatory, and digestive systems are highly conserved in both zebrafish and humans [16,17]. It has recently been shown that hydroxyapatite nanoparticle (HANP) morphology greatly affects acute inflammation in vivo and in vitro [18]. The present study investigates how NP morphology affects fetal development by exposing four different HANPs (dots, long rods, sheets, and fibers) to zebrafish embryos and evaluating their effects on viability and developmental for up to 5 days post-fertilization (dpf).

## 2. Results and Discussion

**Figure 1:**
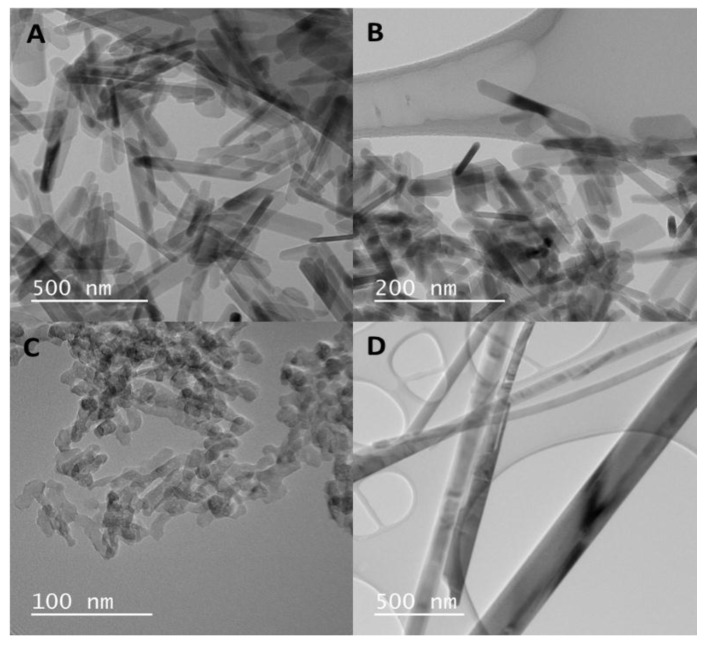
Hydroxyapatite nanoparticle (HANP) transmission electron microscopy (TEM) micrographs of the long rods (**A**), sheets (**B**), dots (**C**), and fibers (**D**).

Four distinct HANP morphologies were synthesized via the hydrothermal method. Transmission electron microscopy (TEM) micrographs revealed that the long rod particles were 20 nm in diameter and 200 nm in length (Figure 1A, Table 1), and the sheet particles were 75 nm in length and 30 nm in width (Figure 1B, Table 1). The dot particles were the smallest in size compared to the other 3 particle morphologies, with an approximate diameter of 15 nm (Figure 1C, Table 1). Although the fiber particles had a width of 60 nm, the particle length ranged from 1 to 4 µm (Figure 1D, Table 1). Phase composition analysis confirmed that all synthesized NPs were composed of HA [18], thereby showing that the effects that the HANPs induce on the zebrafish embryos were due to the particle morphology rather than the particle chemistry.

**Figure 2:**
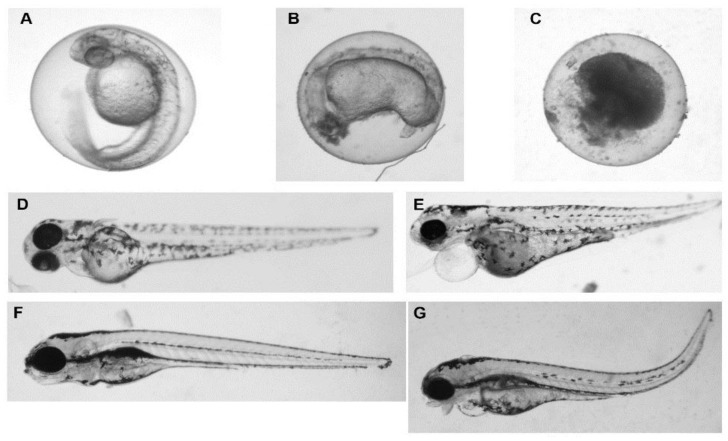
Images of zebrafish after treatment with the HANPs. Image **A** (control, healthy), **B** (malformed), and **C** (dead) embryos at 24 hpf. Image **D** (control, healthy) and Image **E** (malformed) depict zebrafish at 72 hpf, while Image **F** (control, healthy) and **G** (malformed) depict zebrafish at 120 hpf after treatment. These images are representations of pericardial edema and axial curvature malformations that were observed following exposure of the HANPs to the embryos. The observed defect phenotypes were not specific to a particular particle morphology.

Exposure to 40 mg/L of particles resulted in the accumulation of embryonic malformations for all particle types, except long rods, by 5 dpf. Though 17% of the embryos exhibited malformations at day 3, malformations were not present at 5 dpf (Table 2). Malformations, such as pericardial edema and bent tails (Figure 2E,G), were apparent in embryos after 3 days of sheet and long rod particle exposure, and as early as 1 day following dot and fiber particle exposure. Minimal death was observed in response to dot, long rod, and sheets (≤25%). However, fiber particles induced overwhelming toxicity (≤60%) after 1 dpf, and complete toxicity during all subsequent time points.

An increase in the exposure dose to 100 mg/L resulted in increased malformation and toxicity. All embryos suffered from malformations, within 3 days of exposure to sheets, dots, or long rods (Table 3). All embryos exposed to fiber particles died by 1 dpf (as represented by Figure 2C). Despite being exposed to particles for only 24 h, the zebrafish did not recover from the malformations over time. The observed defect phenotypes were not specific to a particular particle morphology. Exposure to particle concentrations of 4 and 20 mg/L did not cause any significant death or developmental defects, and were therefore not shown. It should be noted that the absence of toxicity and mortality at 4 and 20 mg/L suggest that the risk of toxicity from physiologically relevant exposure appear to be quite low.

HANP fiber particles have previously been shown to cause increased oxidative stress, cytotoxicity, and apoptosis in various cell types, e.g., macrophages, neutrophils, and fibroblasts, in vitro as well as increased inflammation in vivo [18]. Oxidative stress has been hypothesized to cause axial curvature [19] in zebrafish. During the early stages of organogenesis, embryos possess a weak antioxidant defense capacity and are, therefore, more sensitive to the teratogenic effects of oxidative compounds. A disequilibrium between reactive oxygen species (ROS) and the embryonic antioxidant defense can therefore, affect the development and function of the zebrafish, giving rise to many different congenital abnormalities such as cardiac edema, growth delay, decrease in pigmentation, and even death [20]. It is likely that the cytotoxicity and oxidative stress that occur following fiber particle exposure in vitro [18], also occur in the zebrafish embryos. Unlike tangled fiber particles, straight fibers, such as the ones used in the current study, cause incomplete uptake and frustrated phagocytosis in cells, leading to mechanical strain and eventual cell death [21]. The mechanical strain that the fiber particles cause could potentially disrupt the chorion of the zebrafish embryos, contributing to the significant death observed. Further studies are needed to confirm whether the nano hydroxyapatite particles disrupt the chorion directly, by contact, or indirectly, as well as evaluate the oxidative stress that the fiber particles cause in zebrafish embryos.

The dot particles caused increased developmental malformations, as compared to the long rod and sheet particles. The dot particles have the highest specific surface area compared to the other particle morphologies [18]. Particles with a high specific surface area are found to be more reactive due to the greater surface area per mass ratio, which can act as a catalyst for reactions between cells and biomolecules [22]. Recent studies have demonstrated that, when exposed to the lungs, particles with higher surface area become trapped, cause clearance impairment and inflammation, eventually leading to fibrosis and cell death [22,23,24]. When exposed to the embryos, the dot particles may translocate through the chorionic pores of the zebrafish and get trapped within the chorionic space. NPs trapped in the chorion can restrict the diffusion of nutrients and oxygen supply to the embryo, causing axial deformities in the tail and spine as well as edema [25,26,27]. The accumulated nanoparticles can also alter the charge, diffusion, and interactions of chorionic proteins such as the hatching enzyme and membrane transporter, leading to interference in the signaling cascades of embryonic development [25,28]. Studies have shown that nanoparticle adsorption to the hatching enzyme causes a delay in hatching [29]. Furthermore, HA particles with properties similar to the dot particles, aggregate together around the membrane transporter protein altering the function of the protein [28]. Taken together, the high specific surface area of the dot particle is most likely the contributing factor causing the observed toxicity and axial deformation. 

## 3. Conclusions

The results of the current study demonstrate that particle morphology greatly affects and influences embryonic development. Although HA is a relatively safe biomaterial, in its nanoparticulate form HA exposure not only induces toxicity, but can cause developmental, and physiological impairments in zebrafish embryos. Increased nanoparticle testing, with an emphasis on particle morphology parameters, should be a required consideration when designing nanomaterials for commercial use.

## 4. Materials and Methods

### 4.1. Particle Preparation and Characterization

The HANPs were fabricated via the hydrothermal method and characterized for phase composition via X-ray diffraction (XRD, Siemens D5000, Texas, TX, USA), and particle morphology via transmission electron microscopy (TEM, Hitachi HF-3300v, Rotkreuz, Switzerland) following the methods of Pujari-Palmer et al. [18].

### 4.2. Zebrafish Embryo Exposure to HANPs

Zebrafish embryos were collected 4 h post-fertilization (hpf) and exposed to the four different HANP solutions at final concentrations of 0, 4, 20, 40, and 100 mg/L at 28.5 °C. Zebrafish embryo development and viability were evaluated for up to 5 dpf using bright-field microscopy (Nikon SMZ1500, Nikon, Tokyo, Japan) Image analysis to determine the percentage of dead and malformed embryos over time, was performed using ImageJ software. All animal experiments were performed in strict accordance with the recommendations of “care and use of laboratory animals of Sweden”. The same embryos (*n* = 6–8) were followed throughout the whole study.

## Figures and Tables

**Table 1:** Physicoproperties of the HANPs. Reproduced and modified from [18]. Copyright Elsevier, 2016.

Particle Morphology	Dimensions	Surface Area
Long Rods	20 nm × 200 nm	71.6
Dots	15 nm × 15 nm	91.5
Sheets	75 nm × 30 nm	58.6
Fibers	60 nm × 1–4 μm	52.7

**Table 2:** Phenotypic observations of zebrafish after treatment with the HANPs at 40 mg/L exposure. The percentage mortality represents mortality with respect to the number of embryos present at each time point, rather than cumulative mortality.

	Sheets		Dots		Long Rods		Fibers		Control	
40 mg/L	Malformed	Dead	Malformed	Dead	Malformed	Dead	Malformed	Dead	Malformed	Dead
Day 1	0%	14%	20%	20%	0%	25%	33%	67%	0%	29%
Day 3	27%	0%	25%	0%	17%	0%	0%	100%	0%	0%
Day 5	33%	0%	0%	17%	0%	0%	0%	100%	0%	0%

**Table 3:** Phenotypic observations of zebrafish after treatment with the HANPs at 100 mg/L exposure. The percentage mortality represents mortality with respect to the number of embryos present at each time point, rather than cumulative mortality.

	Sheets		Dots		Long Rods		Fibers		Control	
100 mg/L	Malformed	Dead	Malformed	Dead	Malformed	Dead	Malformed	Dead	Malformed	Dead
Day 1	0%	25%	0%	18%	0%	18%	0%	100%	0%	29%
Day 3	67%	33%	88%	38%	67%	22%	0%	100%	0%	0%
Day 5	0%	100%	0%	100%	0%	100%	0%	100%	0%	0%
